# Rutin prevents EqHV-8 induced infection and oxidative stress via Nrf2/HO-1 signaling pathway

**DOI:** 10.3389/fcimb.2024.1386462

**Published:** 2024-04-25

**Authors:** Li Chen, Shuwen Li, Wenjing Li, Yue Yu, Qi Sun, Wenjing Chen, Huaqi Zhou, Changfa Wang, Liangliang Li, Meng Xu, Muhammad Zahoor Khan, Yubao Li, Tongtong Wang

**Affiliations:** Liaocheng Research Institute of Donkey High-Efficiency Breeding and Ecological Feeding, Liaocheng University, Liaocheng, China

**Keywords:** Rutin, EqHV-8, antiviral agent, oxidative stress, Nrf2/HO-1 signaling pathway

## Abstract

**Introduction:**

The Nuclear factor erythroid 2-related factor 2 (Nrf2)/heme oxygenase-1 (HO-1) signaling pathway has been extensively studied for its role in regulating antioxidant and antiviral responses. The Equid herpesvirus type 8 (EqHV-8) poses a significant threat to the equine industry, primarily manifesting as respiratory disease, abortions, and neurological disorders in horses and donkeys. Oxidative stress is considered a key factor associated with pathogenesis of EqHV-8 infection. Unfortunately, there is currently a dearth of therapeutic interventions available for the effective control of EqHV-8. Rutin has been well documented for its antioxidant and antiviral potential. In current study we focused on the evaluation of Rutin as a potential therapeutic agent against EqHV-8 infection.

**Methods:**

For this purpose, we encompassed both in-vitro and in-vivo investigations to assess the effectiveness of Rutin in combatting EqHV-8 infection.

**Results and Discussion:**

The results obtained from *in vitro* experiments demonstrated that Rutin exerted a pronounced inhibitory effect on EqHV-8 at multiple stages of the viral life cycle. Through meticulous experimentation, we elucidated that Rutin’s antiviral action against EqHV-8 is intricately linked to the Nrf2/HO-1 signaling pathway-mediated antioxidant response. Activation of this pathway by Rutin was found to significantly impede EqHV-8 replication, thereby diminishing the viral load. This mechanistic insight not only enhances our understanding of the antiviral potential of Rutin but also highlights the significance of antioxidant stress responses in combating EqHV-8 infection. To complement our *in vitro* findings, we conducted *in vivo* studies employing a mouse model. These experiments revealed that Rutin administration resulted in a substantial reduction in EqHV-8 infection within the lungs of the mice, underscoring the compound’s therapeutic promise *in vivo*.

**Conclusion:**

In summation, our finding showed that Rutin holds promise as a novel and effective therapeutic agent for the prevention and control of EqHV-8 infections.

## 1 Introduction

Equid herpesvirus type 8 (EqHV-8), also referred to as asinine herpesvirus 3 (ASH-3), has been implicated in a range of clinical manifestations in equines, including abortions, respiratory diseases, and viral encephalitis. Taxonomically, EqHV-8 belongs to the genus Varicellovirus within the subfamily Alphaherpesvirinae of the family Herpesviridae (Ficorilli et al., 1995; Davison et al., 2009). Notably, donkeys have been identified as the natural hosts for EqHV-8, with the virus originally isolated from the nasal cavity of donkeys in Australia in 1987 (Browning et al., 1988). Furthermore, EqHV-8, specifically the EqHV-8 Wh strain, has been isolated from horses exhibiting fever and nasal discharge in China in 2010 (Liu et al., 2012). In recent years, EqHV-8 has emerged as a cause for concern due to its rapid spread across several countries, leading to significant economic losses in donkey and horse farming operations (Schvartz et al., 2020; Yoon et al., 2022; Wang et al., 2023). Regrettably, effective therapeutic interventions or vaccines for EqHV-8 have remained elusive thus far.

Rutin, a member of the flavonoid class, is widely distributed in nature and has garnered attention for its diverse pharmacological properties, including antioxidant, anti-inflammatory, antiviral, antitumor, and antibacterial effects (Ganeshpurkar and Saluja, 2017; Negahdari et al., 2021; Lai et al., 2023; Miklasinska-Majdanik et al., 2023; de Jesus et al., 2024). Recent research has unveiled Rutin’s broad-spectrum antiviral activity against various viruses (Ninfali et al., 2020). For instance, Rutin has demonstrated antioxidant activity in animals infected with influenza virus, mitigating oxidative damage in the target organs of mice afflicted with influenza virus infection (Savov et al., 2006). Notably, Nrf2, a pivotal transcription factor, plays a central role in orchestrating antioxidant and antiviral responses by regulating the expression of downstream target genes (Seminotti et al., 2021). Concurrently, HO-1, recognized as a cytoprotective protein, assumes a critical role in mediating oxidative stress responses and modulating immune responses (Kovacsics et al., 2017). Significantly, Rutin has recently been found to alleviate oxidative stress induced by ferroptosis in aging chicken small white follicles through the Nrf2/HO-1 pathway (Wu et al., 2023). Furthermore, Rutin has been extensively researched for its medicinal properties, showing promising results across various studies. Initially, its role as an antioxidant was explored, with a notable study by Mohamed et al. demonstrating its efficacy in mitigating the effects of iron oxide nanoparticles in rats (Mohamed et al., 2024). This foundational antioxidant property of Rutin sets the stage for its broader antiviral applications. Han et al., reported Rutin’s antiviral activity against plant viruses such as tobacco mosaic virus (TMV) and cucumber mosaic virus, showcasing its potential beyond antioxidant activity (Han et al., 2015). The scope of Rutin’s antiviral effects was further broadened by research conducted by Agrawal et al. and Chéron et al., which extended its application to combating significant human pathogens, including the replication of SARS-CoV-2, the virus responsible for COVID-19, and norovirus, known for causing gastroenteritis (Chéron et al., 2015; Agrawal et al., 2021). These studies collectively highlight Rutin’s versatile therapeutic potential, from antioxidative to antiviral applications. However, the specific antiviral effects of Rutin and the underlying mechanisms in the context of EqHV-8 infection remain inadequately understood. The present study represents an endeavor to elucidate the anti-EqHV-8 activity of Rutin and explore the potential antiviral mechanisms involved. Our research showed that Rutin exerts a robust anti-EqHV-8 effect in both susceptible cells and a murine model. Furthermore, we have demonstrated that this anti-EqHV-8 effect of Rutin is contingent upon the Nrf2/HO-1-mediated antioxidant stress response pathway. These findings collectively suggest that Rutin holds promise as a potential effective therapeutic agent for the control of EqHV-8 infections.

## 2 Materials and methods

### 2.1 Cells, reagents, viruses, and antibodies

Rabbit kidney cells (RK-13) and Madin-Darby Bovine Kidney (MDBK) cells were purchased from the China Center for Type Culture Collection (CCTCC, Wuhan, China) and maintained in 10% fetal bovine serum (FBS) Minimum Eagle’s medium (MEM) or Dulbecco’s minimal essential medium (DMEM) at 37°C and 5% CO_2_. Rutin was purchased from Shandong Sparkjade Biotechnology Co., Ltd (Jinan, China) and dissolved with DMSO. CoPP (HO-1 inducer) was purchased from Sigma-Aldrich (St. Louis, Missouri, USA). The EqHV-8 isolates were used for this study as follows: SDLC66 (GenBank: MW816102.1), SD2020113 (GenBank: MW822570.1), donkey/Shandong/10/2021 (GenBank: OL856098.1). These EqHV-8 strains were proliferated in MDBK cells, and titrated by a plaque formation assay as previous reported (Li et al., 2023). The anti-EqHV-8-positive serum and mouse anti-gD polyclonal antibody were prepared in our lab. Anti-Nrf2 rabbit pAb, Anti-HO-1 mouse mAb and Anti-α-Tubulin mouse mAb were obtained from Servicebio (Wuhan, China), and horseradish peroxidase (HRP)-labeled goat anti-mouse IgG (H+L) and horseradish peroxidase (HRP)-labeled goat anti-rabbit IgG (H+L) were purchased from Jackson (Lancaster, Pennsylvania, USA).

### 2.2 Cell viability assay

RK-13 and MDBK cells were seeded into 96-well plates (1×10^4^/well) overnight respectively. These cells were incubated with Rutin at various concentrations (10 μM, 20 μM, 40 μM, 80 μM, 160 μM and 200 μM) for 24 h. then, these cells were incubated with the CCK-8 reagent (10 μL/well) for 2 h. The number of viable cells was determined by the EpochTM Microplate spectrophotometer (BioTek, USA) at 450 nm. Finally, these data were analyzed using GraphPad Prism 8.0.

### 2.3 EqHV-8 inhibition assay

RK-13 and MDBK cells were seeded into 12-wells plates respectively and cultured overnight at 37°C and 5% CO_2_. These cells were pre-incubated with Rutin at different concentrations (40 μM, 80 μM, or 160 μM) for 1 h, then infected with EqHV-8 SDLC66(MOI=0.1). The cell samples and cellular supernatant were collected to analyze EqHV-8 replication at 24 h post-infection (hpi) by western blotting and qPCR. Further, the inhibition experiments also performed as follows, the RK-13 and MDBK cells pre-treated with Rutin (160 µM) for 1 h, then infected with EqHV-8 SDLC66 at various MOI (0.1, 0.5, and 1). The cell samples and cellular supernatant were collected to analyze EqHV-8 replication at 24 hpi by qPCR.

### 2.4 Direct inactivation assay and time course analysis of Rutin against EqHV-8

To test whether Rutin could inactivate the EqHV-8, the RK-13 cells were seeded into 12-well cell plates overnight. The EqHV-8 SDLC66 (0.1 MOI, and 0.5 MOI) was incubated with Rutin (160 µM) at 37°C for 1 h, the mixture EqHV-8 and Rutin was incubated in those cells for 1 h. Finally, these cells were harvested to analyze glycoprotein D protein (gD) expression by qPCR and western blotting at 24 hpi. To further determine the stage of the EqHV-8 life cycle interfered with by Rutin, RK-13 cells were cultured into 12-well plates and termed as pre-treated, co-treated, and post-treated with Rutin (160 µM) relative to the EqHV-8 (MOI=0.1) inoculation groups. After 24 h, the cells were collected to examine the gD expression of EqHV-8 by qPCR and western blotting.

### 2.5 Real-time quantitative PCR

The total RNA of cells samples was extracted by TriQuick Reagent (Solarbio, China), and 1 μg of the total RNA was converted to cDNA using the PrimeScript™ RT Master Mix kit (Takara, Japan) according to the manufacturer’s instructions. The qPCR was performed using a StepOnePlus^®^ Real-Time PCR applied biosystems (Thermo, USA), the gene expressions of gD, Nrf2, and HO-1 were detected and normalized against those of GADPH using the 2^−ΔΔCt^ threshold cycle (CT) method as previously described (Li et al., 2022). All primers are listed in **Table 1**. The absolute quantification (qPCR) was performed to detect the copy number of EqHV-8 genome DNA as described previously (Li et al., 2023). The recombinant plasmid of pMD18-T-*ORF72* served as the template to calculate the EqHV-8 genome DNA copies.

**Table 1:** The primers in this study.

Primers	Primer sequences (5´-3´)
HO-1-F	AGTTCATGAAGAACTTTCA
HO-1-R Nrf2-F Nrf2-R	TACCAGAAGGCCATGTCC ATTCAATGATTCTGACTCTG CGTATCCCCAGAAGAATGTA
ORF72-F	CCCACGTGTGCAACGCCTAT
ORF72-R	ATACAGTCCCGAGGCAGAGT
EHV8-gD-F	GATGCCAAACCGAATCAGCC
EHV8- gD-R	TAGGCGAGTCAAGCCGTTTT
GAPDH-F	CCTTCCGTGTCCCTACTGCCAAC
GAPDH-R	GACGCCTGCTTCACCACCTTCT
siRNA-HO-1	GGTCCTCACACTCAGCTTT
siRNA-Nrf2	CTCCTTAAGAAGCAACTCA

### 2.6 Western blotting

All cell samples were harvested, and lysed with NP-40 lysis buffer (Solarbio, China) and mixed with 5× sample loading buffer. Next, the proteins were loaded onto 12% sodium dodecyl sulfate–polyacrylamide gel electrophoresis (SDS–PAGE) gels with equal amounts and transferred onto polyvinylidene fluoride (PVDF) membranes as described previously (Wang et al., 2019). The PVDF membranes were blocked with 2.5% skimmed milk and incubated with anti-Nrf2, anti-HO-1 mAb, anti-α-Tubulin mAb (Servicebio, China), or anti-EqHV-8 gD polyclonal antibody. HRP-conjugated goat anti-mouse or goat anti-rabbit IgG was used as the secondary antibody. Then, the protein band signals were detected by ChemiDoc XRS imaging system (Bio-Rad) with an Enhanced Chemiluminescent (ECL) kit (Bio-Rad, USA).

### 2.7 ROS, MDA, SOD, and GSH-PX detection

Dichlorofluorescein (DCF) ROS detection kits were purchased from Beyotime Biotechnology (Nanjing, China), MDA detection kit, SOD detection kit, and GSH-PX detection kit were obtained from Jiancheng Bioengineering Institute (Nanjing, China). These kits were used to determine the expression of ROS, MDA, SOD and GSH-PX in RK-13 cells according to the manufacturer instructions respectively. ROS production were observed using Leica DMi8. Meanwhile, the fluorescent intensity was measured by Spark microplate reader (Tecan, Switzerland).MDA, SOD and GSH-PX generation were detected by Epoch microplate spectrophotometer (BIOTEK, USA). The levels were normalized to protein concentrations measured by Pierce™ BCA Protein Assay Kit (Thermo, Massachusetts, USA).

### 2.8 RNAi assay

The siRNAs targeting the Nrf2 and HO-1 genes were synthesized by Ribo Biotechnology Co., Ltd. (Guangdong, China). The siRNA knockdown assay was performed as previously described (Li et al., 2022). In brief, the RK-13 cells were transfected with these siRNAs according to the Lipo 6000 protocol (Beyotime, China), respectively, and non-targeting siRNA served as a negative control. The total RNA of these cells was extracted using TRizol reagent. The mRNA levels of the Nrf2, HO-1, and EqHV-8 gD gene were determined by qPCR and normalized against those of GAPDH by the 2^−ΔΔCt^ threshold cycle (CT) method. All the samples were performed in three independent experiments.

### 2.9 Animal experiments

The EqHV-8 challenge assay was performed as previously described (Wang et al., 2022a). Briefly, fifteen specific pathogen-free, 6-week-old male BALB/c mice were obtained from the Pengyue Experimental Animal Breeding Co., Ltd (Jinan, China) and divided into three groups randomly (n =5 mice/group). Group 1 mice were inoculated intranasally with 100 μL of DMEM and served as Mock group, Group 2 mice were inoculated intraperitoneally with 100 μL of DMSO solution (at final concentration 0.1%), and Group 3 were incubated Rutin (35 mg/kg) intraperitoneally. Mice of Group 2, Group 3 were challenged intranasally with 100 μL of EqHV-8 SDLC66 (1×10^5^ Pfu/mice), then inoculated intraperitoneally with DMSO or Rutin at -1, 1 and 2 day post infection (dpi). Mice in each group were housed separately to prevent cross-infection. Notably, the incubation of EqHV-8, DMEM, Rutin or DMSO into mice was performed under deep anesthesia by Zoletil 50 (Virbac, Nice, France). The clinical symptoms of mice were monitored daily. Blood was collected at 3, 5, 7 dpi, and the lungs of all mice were obtained via euthanizing by cervical dislocation at 8 dpi, which was used to assess viremia and histopathology change.

#### 2.9.1 Viremia detection

The viral DNA of serum was extracted by viral DNA extraction kit (Omega Bio-Tek, USA) according to the manufacturer’s protocol. The EqHV-8 genome copies in the serum were calculated by absolute qPCR based on part *ORF72* gene as previously described (Li et al., 2023).

#### 2.9.2 Histopathology evaluation

The hematoxylin and eosin (HE) assay were performed to evaluate histopathological changes in lungs from different groups mice at 8 dpi as previously described (Wang et al., 2022a). Briefly, lungs of each group were fixed into 10% formalin solution, underwent dehydration by alcohol, transparentized in xylene, and then embedded in paraffin wax. further sliced in a microtome (Leica, Nussloch, Germany) to 4 µm, affixed onto slides, followed by deparaffinization, clearance, then, diluted with 75% and 95% alcohols, and hematoxylin solution stains. The tissue slices were differentiated, using 95% or 100% alcohol directly after the eosin stain, and, after cleared, placed on a coverslip to be observed by light microscopy.

### 2.10 Statistical analysis

Statistical analysis was performed using GraphPad Prism software. Differences among the groups were analyzed by unpaired Student’s t-test. Significance is indicated as follows: *, P < 0.05; **, P < 0.01; and ***, P < 0.001.

## 3 Results

### 3.1 Chemical structure and cytotoxicity of Rutin

**Figure 1:**
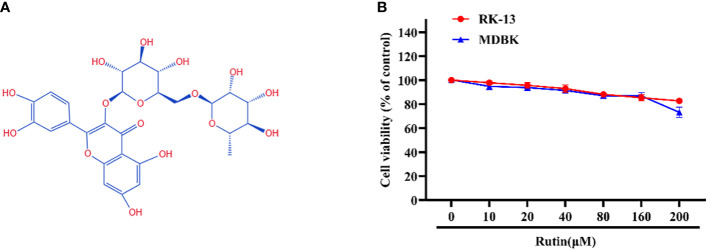
Chemical structure and cytotoxicity of Rutin; **(A)** The chemical structure of Rutin. **(B)** The cytotoxicity of Rutin in RK-13 and MDBK cells. Cells were seeded in 96-well cell plates and treated with different concentrations of Rutin (10 μM, 20 μM, 40 μM, 80 μM, 160μM and 200 μM) for 24 h. The viability of cells was determined using the CCK-8 assay. These data are representative of three independent experiments.

The chemical structure of Rutin is illustrated in **Figure 1A**. The cytotoxicity of Rutin in RK-13 and MDBK cells was determined using the CCK-8 kit. Our data showed that the maximum safe concentration of Rutin in these cells is 160 μM (**Figure 1B**).

### 3.2 Rutin decreases EqHV-8 replication in susceptible cell

**Figure 2:**
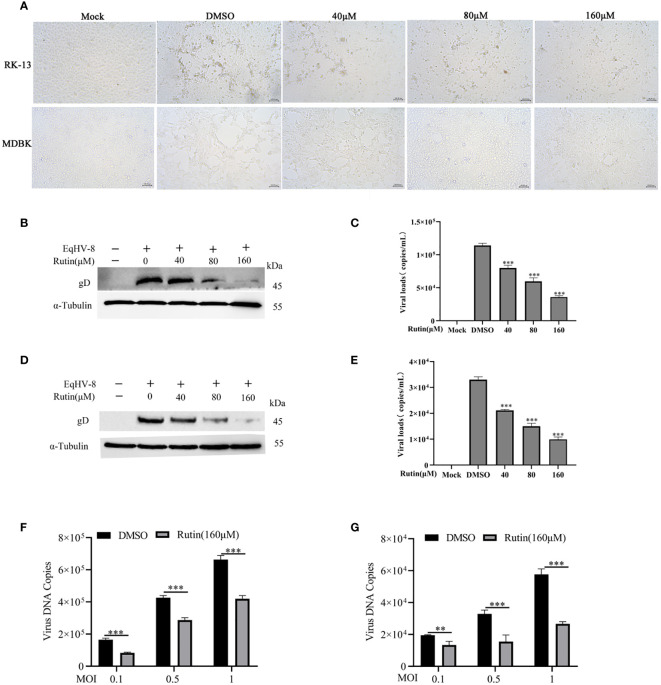
Anti-EqHV-8 activity of Rutin in susceptible cells. RK-13 cells were pre-incubated with different concentrations of Rutin for 1 h and afterward infected with EqHV-8 SDLC66 at 0.1 MOI. The CPE of different groups were observed and imaged by Leica DMi8 microscope **(A)**. The production of gD protein was analyzed by western blotting **(B)**, and the progeny virus’s generation was measured by qPCR **(C)**. MDBK cells were pretreated with Rutin using the same protocol, and the EqHV-8 replication was determined by western blotting **(D)**, and qPCR **(E)**. RK-13 **(F)** and MDBK **(G)** cells pre-treated with Rutin (160 µM) for 1 h, then infected with EqHV-8 SDLC66 at various MOI (0.1, 0.5, and 1). The progeny virus generation was detected by qPCR at 24 hpi. α-Tubulin as a loading control. The data shown are representatives from three independent experiments and subjected to unpaired student’s t-tests. **P < 0.01 (compared with DMSO-treated cells), ***P < 0.001 (compared with DMSO-treated cells). These data are representative of three independent experiments.

To assess the potential anti-EqHV-8 activity of Rutin *in vitro*, RK-13 and MDBK cells were pre-treated with varying concentrations of Rutin (0 μM, 40 μM, 80 μM, and 160 μM) for 1 h and subsequently infected with EqHV-8 SDLC66 at 0.1 MOI. The CPE of different concentrations Rutin treated group were observed and imaged at 24 hpi (**Figure 2A**). Then, both cellular samples and cellular supernatants were subjected to analysis for EqHV-8 replication through western blotting and qPCR. As depicted in **Figure 2B**, Rutin exhibited a dose-dependent reduction in gD protein expression in RK-13 cells. Consistent with the protein analysis results, the production of progeny viruses in the Rutin-treated group showed a significant decrease compared to the group treated with DMSO, as illustrated in **Figure 2C**. Similar outcomes were observed in MDBK cells, as shown in **Figures 2D** and **2E**. Further, RK-13 and MDBK cells were pre-treated with Rutin (0 μM, or 160 μM) for 1 h and subsequently infected with EqHV-8 SDLC66 at different MOI (0.1, 0.5 and 1MOI). As illustrated in **Figures 2F, G**, Rutin reduces progeny virus generation in RK-13 and MDBK cells.

### 3.3 Rutin inhibits other EqHV-8 strains’ infection

**Figure 3:**
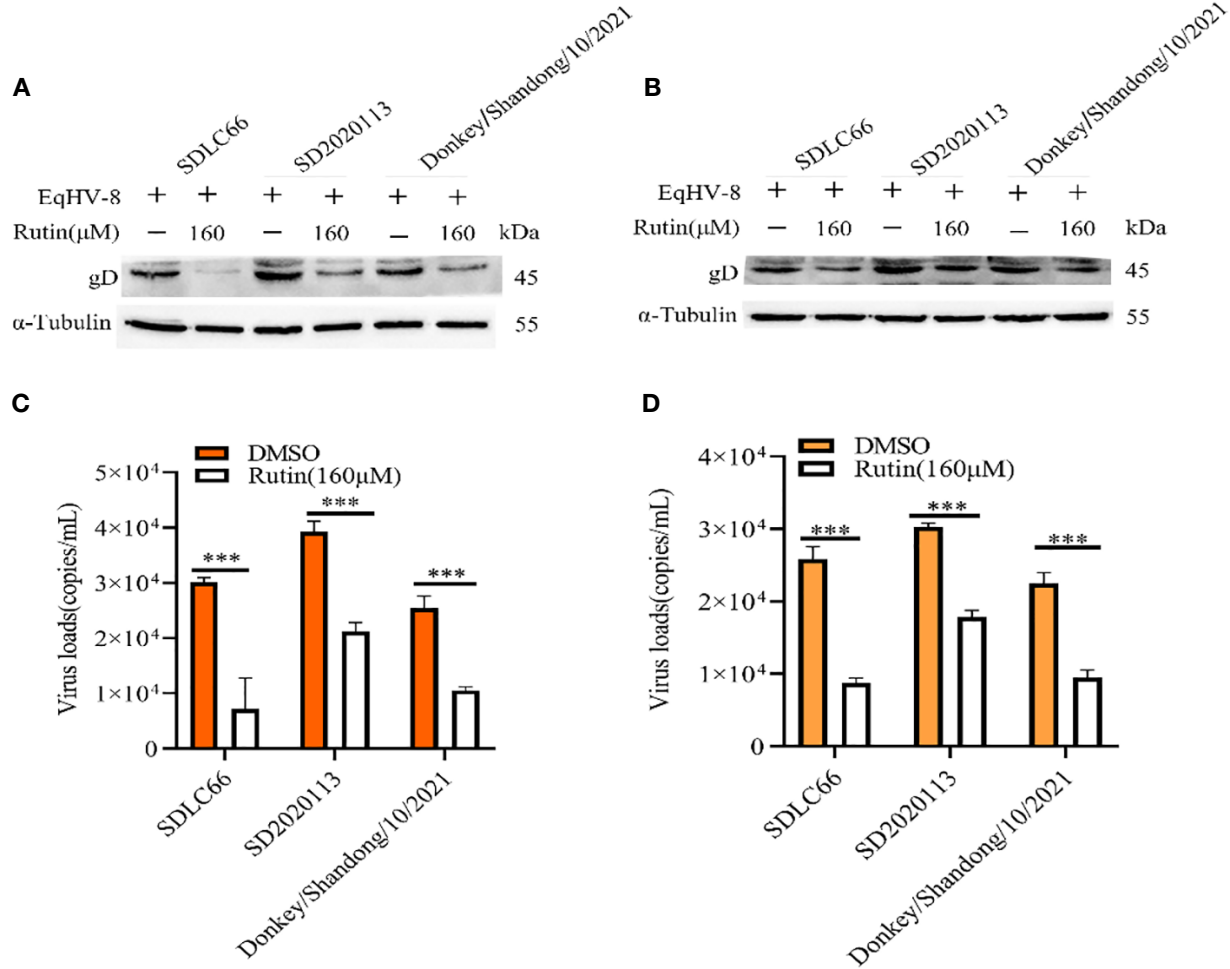
Rutin inhibits other strains of EqHV-8 infection in susceptible cells. The RK-13 and MDBK cells were incubated with the presence or absence of Rutin (160 µM) for 1 h, then infected with SDLC66, SD2020113, donkey/Shandong/10/2021 strains 1 h at 37°C, respectively. The EqHV-8 replication at 24 hpi was evaluated by western blotting in RK-13 cells **(A)** or MDBK cells **(B)**. The progeny virus generation was tested by qPCR in RK-13 cells **(C)** or MDBK cells **(D)**. ***, P < 0.001 compared with DMSO-treated cells challenged with the same virus. These data are representative of three independent experiments.

We further assessed the antiviral activity of Rutin against other EqHV-8 strains, including SD2020113 and donkey/Shandong/10/2021, in both RK-13 and MDBK cells. EqHV-8 replication was evaluated using western blotting and qPCR. The results demonstrated a significant decrease in gD protein expression in Rutin-treated RK-13 cells compared to the DMSO-treated group, as depicted in **Figure 3A**. Similar outcomes were observed in MDBK cells, as illustrated in **Figure 3B**. In accordance with the western blotting results, the production of EqHV-8 progeny was also significantly reduced in the Rutin-treated group in both RK-13 (**Figure 3C**) and MDBK cells (**Figure 3D**). Our data collectively indicate that Rutin exhibits broad-spectrum anti-EqHV-8 infection activity.

### 3.4 Rutin reduces EqHV-8 infection at different stages

**Figure 4:**
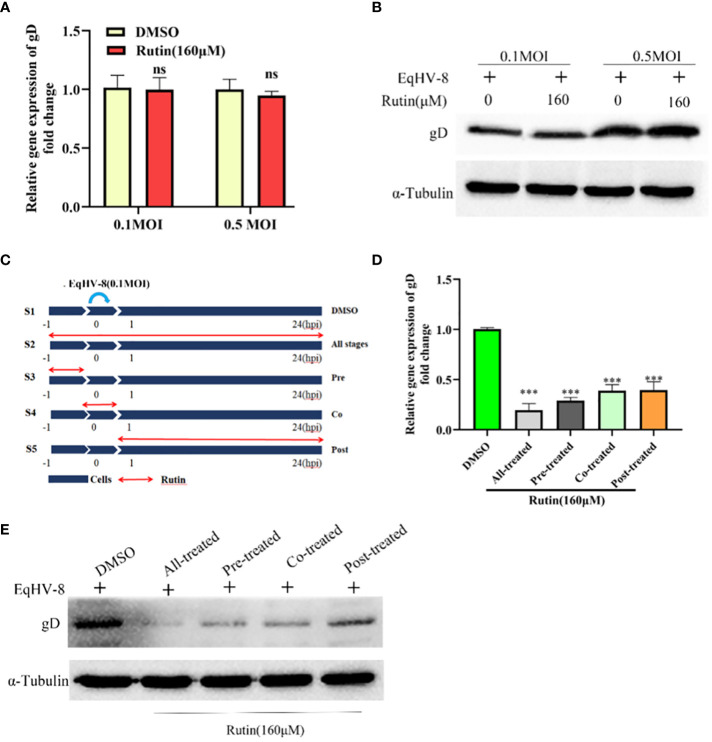
Rutin inhibits EqHV-8 infection at multiple stages of the virus life cycle. **(A, B)** Rutin could not inactivate EqHV-8 directly. EqHV-8 SDLC66 with different dose age (0.1 MOI or 0.5 MOI) pre-incubated with Rutin (160 µM) for 1 h at 37°C, then the mixture was added into RK-13 cells. qPCR and western blotting were used to evaluate gD protein expression at 24 hpi. **(C)** Schematic diagram of Rutin-treated cells, the RK-13 cells were infected with 0.1 MOI EqHV-8 SDLC66 and treated with Rutin (160 µM) at different times of infection, including all-stage treatment, pre-treatment, co-treatment, and post-treatment. The expression of gD protein was determined by qPCR **(D)** and western blotting **(E)** at 24 hpi. GAPDH served as an internal control. α-Tubulin acts as a loading control. The data shown are representatives from three independent experiments subjected to unpaired Student’s t-tests. ***P < 0.001 (compared with DMSO-treated group cells). These presented data is the representative of three independent experiments.

To ascertain whether EqHV-8 could be effectively inactivated by Rutin directly, we conducted experiments employing RK-13 cells. These cells were subjected to incubation with a mixture containing Rutin at a concentration of 160 μM and EqHV-8 at varying dosages, specifically 0.1 MOI and 0.5 MOI. As illustrated in **Figures 4A, B**, there was no discernible alteration in gD expression between the DMSO-treated group and the Rutin-treated group. This observation implies that Rutin does not possess the capability to directly inactivate EqHV-8.

To further elucidate the specific stage of the EqHV-8 replication cycle that may be affected by Rutin, we conducted a time-of-addition experiment, as depicted in **Figure 4C**. In this experiment, S1 represents the DMSO-treated group, which serves as the control. S2 signifies Rutin treatment administered at all stages of EqHV-8 infection. S3 represents the Rutin pre-treated group, while S4 designates the Rutin and EqHV-8 co-treated group. Lastly, S5 denotes the Rutin post-treated group. Subsequently, RK-13 cells were collected at 24 hpi to measure the expression of the gD protein and assess the production of progeny viruses. The results revealed a noteworthy decrease in gD expression, both at the transcriptional and protein levels, in the S2, S3, S4, and S5 groups when compared to the S1 control group (**Figures 4D, E**). These findings collectively indicate that Rutin exerts inhibitory effects on EqHV-8 infection at multiple stages within the virus’s life cycle.

### 3.5 Rutin alleviates EqHV-8−stimulated oxidative stress via Nrf2/HO-1 axis

**Figure 5:**
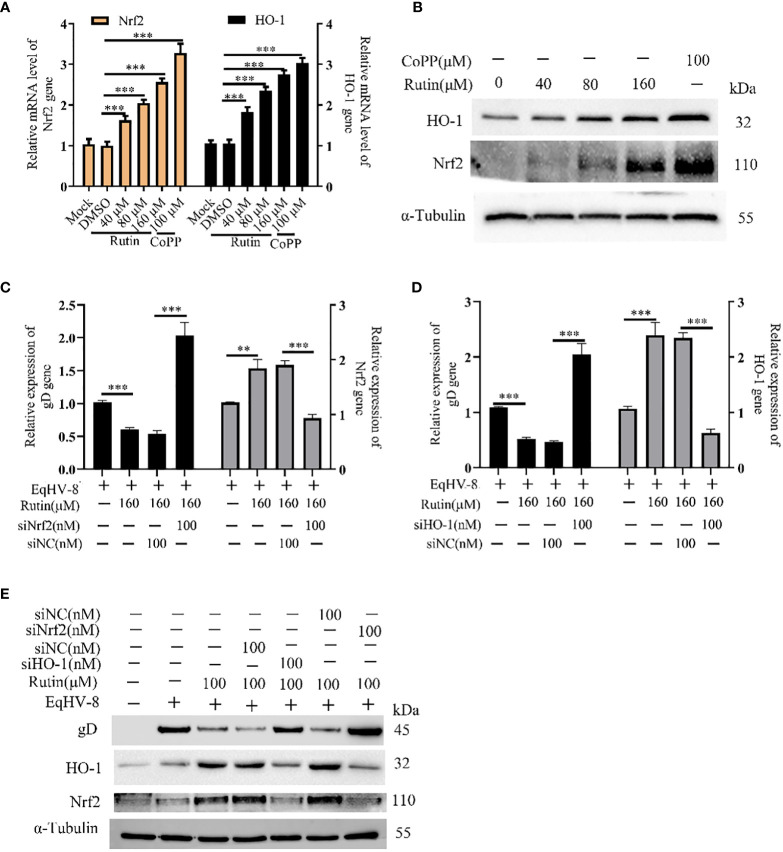
Rutin decrease EqHV-8 infection via Nrf2/HO-1 activation. The RK-13 cell was treated by Rutin with different concentrations (40 μM, 80 μM, and 160 μM) or CoPP (100 μM) for 24 h, then collected to analyze Nrf2/HO-1 expression by RT-qPCR **(A)** and western blotting **(B)** ***, P < 0.001, compared with DMSO treated cells. **(C–E)** The RK-13 cell were transfected by siNrf2 or siHO-1 for 12 h, and treated by Rutin (160 μM) for 1 h, the cells were infected with 0.1 MOI EqHV-8 SDLC66 for 1 h, the Nrf2, HO-1, and gD expression were determined by qPCR and western blotting at 24 hpi. GAPDH served as an internal control. α-Tubulin acts as a loading control. **, P < 0.01, ***, P < 0.001, compared with DMSO or siNC treated cells. These data are representative of three independent experiments.

In order to investigate whether Rutin has the potential to modulate Nrf2/HO-1 expression, we subjected RK-13 cells to various dosages of Rutin treatment for a duration of 24 h. Subsequently, the cells were harvested for the analysis of Nrf2/HO-1 expression using both qPCR and Western blotting techniques. As depicted in **Figure 5A**, the mRNA levels of Nrf2 and HO-1 exhibited a significant increase in response to Rutin treatment when compared to the DMSO-treated group. Concurrently, the changes observed in gD protein levels were consistent with the alterations observed at the mRNA level, as illustrated in **Figure 5B**. To further explore the anti-EqHV-8 effect of Rutin in association with Nrf2/HO-1 activation, we conducted experiments involving RK-13 cells. These cells were pre-incubated with Rutin, siNrf2, or siHO-1, followed by infection with EqHV-8. Subsequently, the expression of Nrf2, HO-1, and gD genes was analyzed. Our results clearly demonstrated that Rutin reduced the expression of the gD gene by inducing Nrf2 and HO-1. This effect was reversed upon treatment with siNrf2 or siHO-1, as observed at both the transcriptional level (**Figures 5C, D**) and the protein level (**Figure 5E**).

**Figure 6:**
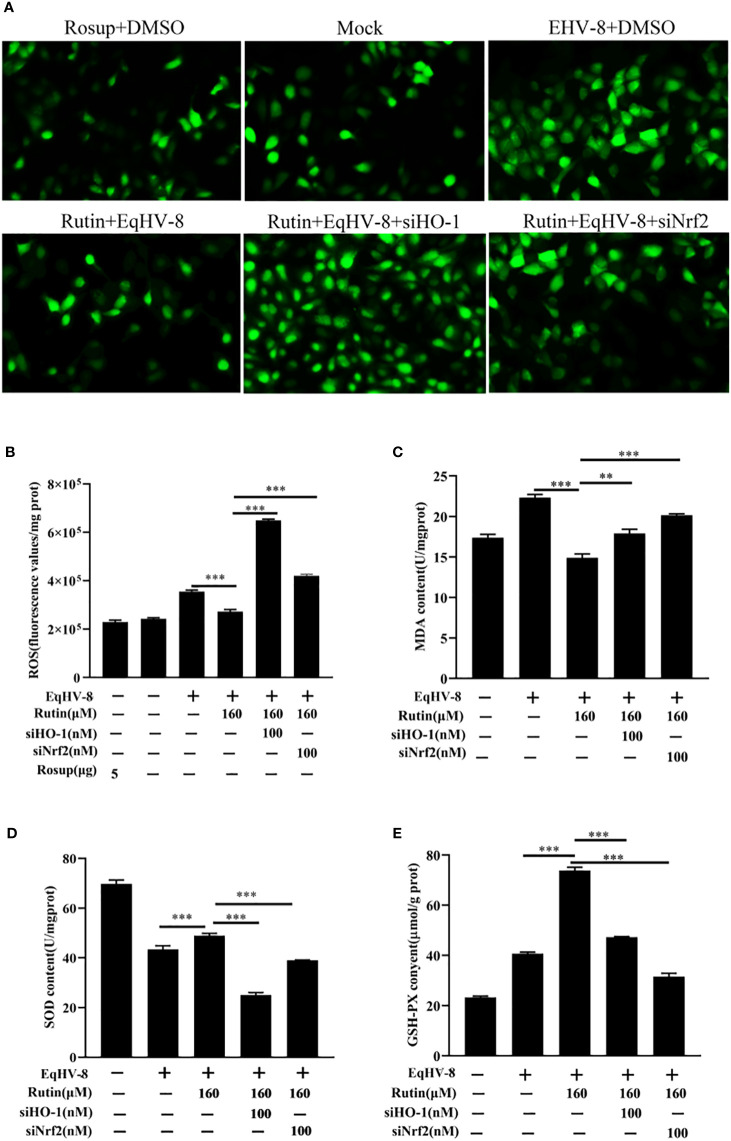
Rutin lessen oxidative stress induced by EqHV-8 infection. The RK-13 cells were transfected by siNrf2 or siHO-1 for 10 h, and pre-incubated with Rutin (160 µM) for 1 h, then infected with EqHV-8 SDLC66 0.1 MOI for 1 h, and ROS generation in RK-13 cells at 24 hpi was determined using DCFH-DA assay. The fluorescence was observed using Leica DMi8 **(A)** and measured by a Spark microplate reader **(B)**. The effects of Rutin on MDA **(C)**, SOD **(D)**, and GSH **(E)** levels in RK-13 cells were also detected at 24 hpi. **, P < 0.01,***, P < 0.001. These data are representative of three independent experiments.

In order to further investigate and analyze whether the anti-EqHV-8 activity of Rutin is associated with its antioxidant properties, we subjected RK-13 cells to pre-treatment with varying concentrations of Rutin, and infected with EqHV-8. As depicted in **Figures 6A, B**. EqHV-8 infection increases significantly the ROS generation in DMSO-treated group. However, Rutin treatment obviously attenuates the ROS production in EqHV-8 infected cells. Subsequently, the levels of MDA within the RK-13 cells were quantified using MDA detection kit. Our findings revealed that EqHV-8 infection led to a notable increase in MDA levels, while Rutin treatment effectively reduced MDA levels in EqHV-8 infected cells when compared to the DMSO-treated group (as shown in **Figure 6C**). In contrast, Rutin treatment also resulted in the upregulation of other important biomarkers, specifically SOD and GSH-PX expression, as illustrated in **Figures 6D, E**. Importantly, these effects of Rutin were found to be reversible upon the administration of specific siHO-1 or siNrf2. Collectively, our data strongly suggest that the anti-EqHV-8 activity of Rutin is largely contingent upon the Nrf2/HO-1-mediated antioxidant stress response.

### 3.6 Rutin inhibits EqHV-8 infection *in vivo*

**Figure 7:**
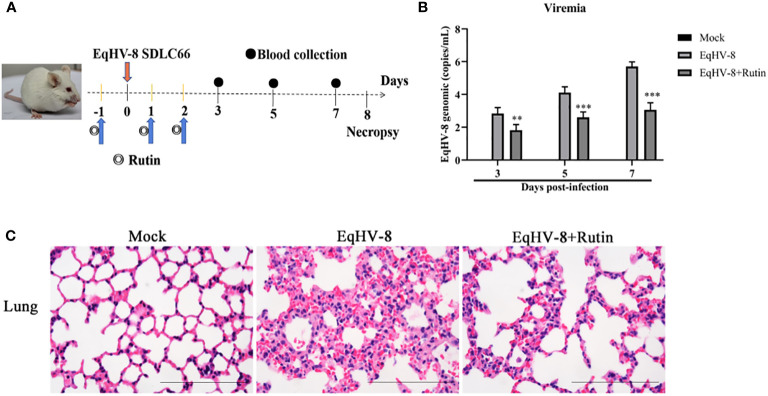
Rutin has antiviral activity against EqHV-8 *in vivo* analysis. **(A)** The pattern diagram of the animal experiments. **(B)** Viral load in sera of mice from each group were measured by qPCR. The results are presented as the mean ± SD (error bars). **P<0.01, ***P<0.001. **(C)** Representative images of hematoxylin and eosin (H&E) in the lungs derived from mice in indicated groups. Bar, 100 µm.

To further corroborate the potential antiviral effect of Rutin against EqHV-8 infection *in vivo*, we conducted an assessment of viremia and histopathological lesions in a mouse model. **Figure 7A** provides an overview of the experimental design. In the serum, Rutin treatment led to a significant reduction in EqHV-8 copies when compared to the group infected solely with EqHV-8, as depicted in **Figure 7B**. Microscopic examination of lung lesions in the group infected only with EqHV-8 revealed several pathological features, including thickened alveolar septa, an increased number of inflammatory cells, hyperemia, and hemorrhage of alveolar septa. Rutin administration conspicuously ameliorated lung damage in the mouse model, as illustrated in **Figure 7C**.

## 4 Discussion

In recent years, the emergence of EqHV-8 in equids, specifically donkeys and horses, has garnered significant attention due to its detrimental impact on the equine industry. EqHV-8 has been identified as a causative agent of abortion, respiratory diseases, and neurological disorders in these animals, resulting in substantial economic losses within the industry (Liu et al., 2012; Garvey et al., 2018). Notably, our previous research endeavors have elucidated the severity of EqHV-8 infection, particularly in donkeys from large-scale farms, emphasizing its high prevalence in China and its profound threat to the donkey industry (Wang et al., 2022a; Wang et al., 2022b; Wang et al., 2023). Despite the devastating consequences of EqHV-8 infection, an effective intervention strategy, such as antiviral drugs or vaccines, has remained elusive until the present day. This knowledge gap prompted our investigation into potential therapeutic options against EqHV-8. Our current study has provided compelling evidence that Rutin, a naturally occurring flavonoid, exhibits potent antiviral activity against EqHV-8 infection through the modulation of the Nrf2/HO-1-mediated antioxidant stress response pathway.

**Figure 8:**
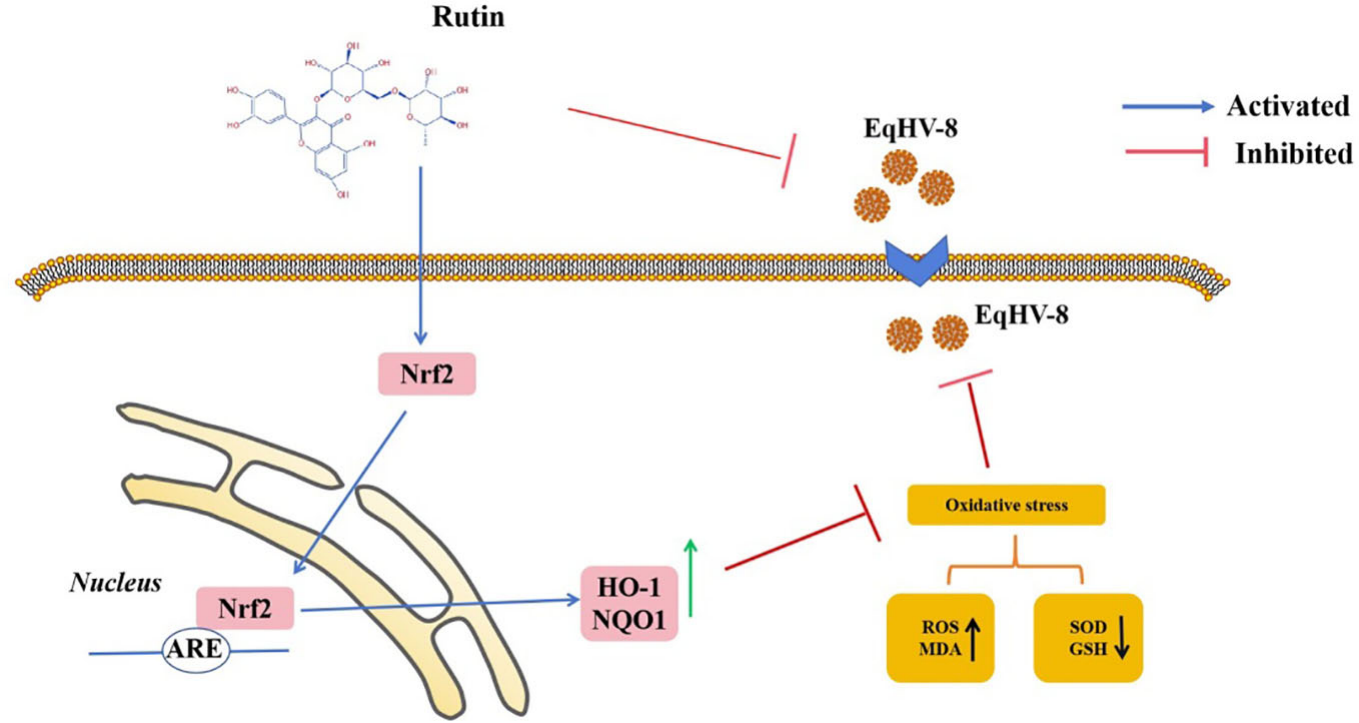
Schematic exhibition that Rutin decrease EqHV-8 infection. Rutin inhibits EqHV-8 infection at multiple stages to susceptible cell. Noteworthy, Rutin treatment also activates Nrf2 and its downstream HO-1 and NQO1, which leads to decreases oxidative stress with a suppression of ROS and MDA, and an increase of GSH and SOD in host cellular, resulting in an inhibition of EqHV-8 replication.

In the realm of animal studies, there exists a robust body of research indicating that herpesvirus infection imposes a noteworthy physiological burden in the form of oxidative stress (Costantini et al., 2018). Specifically, their investigation highlights the correlation between herpesvirus infection and an elevated generation of ROS, resulting in molecular oxidative damage. Furthermore, this pathological process is accompanied by discernible alterations in both non-enzymatic and enzymatic antioxidant defense mechanisms (Sebastiano et al., 2016). Rutin has garnered increasing attention in recent years due to its diverse biological effects, encompassing antioxidant, anti-inflammatory, antidiabetic, antimicrobial, and anti-cancer properties (Perk et al., 2014; Budzynska et al., 2019; Khan et al., 2020; Mazik, 2022). Moreover, Rutin has demonstrated broad-spectrum antiviral activity against several viral pathogens, including influenza virus, hepatitis C virus, norovirus, enterovirus A71, and even the notorious SARS-CoV-2 (Lin et al., 2012; Chéron et al., 2015; Zakaryan et al., 2017; Ling et al., 2020; Mazik, 2022). Our investigation extends this repertoire by showcasing the inhibitory effects of Rutin on EqHV-8 infection. To delve into the specifics of our findings, Rutin was found to significantly reduce EqHV-8 infection in RK-13 and MDBK cells in a dose-dependent manner, as illustrated in **Figure 2**. Subsequent experiments con-firmed the broad-spectrum antiviral activity of Rutin against various EqHV-8 strains, as depicted in **Figure 3**. Furthermore, our research elucidated that Rutin exerts its antiviral effects by disrupting multiple stages of the EqHV-8 life cycle, as evidenced in **Figure 4**. Of particular interest is our discovery that Rutin plays a pivotal role in reducing the oxidative stress induced by EqHV-8. Nrf2/HO-1 has been reported to play a critical mediator in regulating antioxidative and antiviral immune response (Loboda et al., 2016; Ma et al., 2024). Previous studies have shown that Rutin via Nrf2/HO-1signaling pathway relieve oxidative stress (Mihic et al., 2022; Wu et al., 2023; Lee et al., 2024). In typical physiological conditions, the Nrf2 is primarily located within the cellular cytoplasm and forms a complex with its inhibitory partner, Kelch-like ECH-associated protein 1 (Keap1) (Bellezza et al., 2018; Abdul-Muneer, 2023). However, when the cellular environment encounters an elevated presence of ROS, the Keap1-Nrf2 complex undergoes dissociation, leading to the translocation of Nrf2 from the cytoplasm into the cellular nucleus. Once in the nucleus, Nrf2 exerts its regulatory function by promoting the transcription of genes that contain antioxidant response elements (ARE) (NQO1, HO-1), which are crucial components of the antioxidant response system. Furthermore, upon regulation, The products of ARE-linked genes remarkable counteract and neutralize ROS-induced oxidative damage (**Figure 8**) (Tonelli et al., 2018; Han et al., 2019). Our cur-rent findings showed this effect of Rutin may attributed to its the regulation of the Nrf2/HO-1 signaling pathway, as demonstrated in **Figures 5**, **6**, and **8**. This mechanistic insight underscores the multifaceted nature of Rutin’s antiviral action against EqHV-8, linking it to the modulation of critical cellular pathways involved in the antioxidant response. Moreover, our study extended beyond *in vitro* cell culture models to validate the protective effect of Rutin against EqHV-8 infection in a mouse model. As depicted in **Figures 7B, C**, Rutin administration effectively mitigated viremia and reduced lung damage induced by EqHV-8 in this *in vivo* setting, providing further support for its therapeutic potential.

## 5 Conclusions

In conclusion, this study provides compelling evidence for Rutin’s potential as an effective therapeutic agent against EqHV-8 infections. Rutin’s ability to inhibit viral replication and alleviate oxidative stress through the Nrf2/HO-1 pathway highlights its promise as a candidate for future anti-EqHV-8 drug development. This research represents a significant step toward addressing the challenges posed by EqHV-8 in the equine industry. However, further investigations are warranted to elucidate the detailed molecular mechanisms involved in Rutin’s antiviral activity and its potential application as a veterinary medicine.

## Data availability statement

The original contributions presented in the study are included in the article/supplementary material. Further inquiries can be directed to the corresponding authors.

## Ethics statement

The animal study was approved by Animal Welfare and Ethics Committee of Institute of Animal Science, Liaocheng University (protocol number LC2023-09). The study was conducted in accordance with the local legislation and institutional requirements.

## Author contributions

LC: Software, Resources, Investigation, Formal analysis, Data curation, Writing – review & editing, Writing – original draft, Methodology. SL: Writing – review & editing, Writing – original draft, Methodology, Investigation, Formal analysis, Data curation. WL: Software, Writing – review & editing, Data curation. YY: Data curation, Methodology, Formal analysis, Validation, Software, Writing – review & editing. QS: Writing - review & editing, Data cuartion, Methodology, Formal analysis, Validation. WC: Investigation, Writing – review & editing, Data curation. HZ: Software, Writing – review & editing, Investigation, Data curation. CW: Writing – review & editing, Visualization, Validation, Project administration, Conceptualization. LL: Writing – review & editing, Software, Investigation, Data curation. MX: Writing – review & editing, Investigation, Data curation. MK: Writing – original draft, Validation, Supervision, Methodology, Writing – review & editing. YL: Writing – review & editing, Writing – original draft, Visualization, Validation, Supervision, Resources, Project administration, Methodology, Investigation, Funding acquisition, Conceptualization. TW: Writing – review & editing, Writing – original draft, Visualization, Validation, Supervision, Resources, Project administration, Methodology, Investigation, Funding acquisition, Formal analysis, Conceptualization.

## References

[B1] Abdul-Muneer P. M. (2023). Nrf2 as a potential therapeutic target for traumatic brain injury. J. Integr. Neurosci. 22, 81. doi: 10.31083/j.jin2204081 37519172

[B2] Agrawal P. K. Agrawal C. Blunden G. (2021). Rutin: A potential antiviral for repurposing as a SARS-CoV-2 main protease (Mpro) inhibitor. Natural Product Commun. 16, 1934578X21991723. doi: 10.1177/1934578X21991723

[B3] Bellezza I. Giambanco I. Minelli A. Donato R. (2018). Nrf2-Keap1 signaling in oxidative and reductive stress. Biochim. Biophys. Acta Mol. Cell Res. 1865, 721–733. doi: 10.1016/j.bbamcr.2018.02.010 29499228

[B4] Browning G. F. Ficorilli N. Studdert M. J. (1988). Asinine herpesvirus genomes: comparison with those of the equine herpesviruses. Arch. Virol. 101, 183–190. doi: 10.1007/BF01310999 2845891

[B5] Budzynska B. Faggio C. Kruk-Slomka M. Samec D. Nabavi S. F. Sureda A.. (2019). Rutin as neuroprotective agent: from bench to bedside. Curr. Med. Chem. 26, 5152–5164. doi: 10.2174/0929867324666171003114154 28971760

[B6] Chéron N. Yu C. Kolawole A. O. Shakhnovich E. I. Wobus C. E. (2015). Repurposing of rutin for the inhibition of norovirus replication. Arch. Virol. 160, 2353–2358. doi: 10.1007/s00705-015-2495-y 26112762

[B7] Costantini D. Seeber P. A. Soilemetzidou S. E. Azab W. Bohner J. Buuveibaatar B.. (2018). Physiological costs of infection: herpesvirus replication is linked to blood oxidative stress in equids. Sci. Rep. 8, 10347. doi: 10.1038/s41598-018-28688-0 29985431 PMC6037783

[B8] Davison A. J. Eberle R. Ehlers B. Hayward G. S. McGeoch D. J. Minson A. C.. (2009). The order herpesvirales. Arch. Virol. 154, 171–177. doi: 10.1007/s00705-008-0278-4 19066710 PMC3552636

[B9] de Jesus L. B. Frota A. F. de Araujo F. M. de Jesus R. L. C. Costa M. F. D. de Vasconcelos D.. (2024). Effect of the flavonoid rutin on the modulation of the myenteric plexuses in an experimental model of Parkinson's disease. Int. J. Mol. Sci. 25, 1037. doi: 10.3390/ijms25021037 38256111 PMC10815896

[B10] Ficorilli N. Studdert M. J. Crabb B. S. (1995). The nucleotide sequence of asinine herpesvirus 3 glycoprotein G indicates that the donkey virus is closely related to equine herpesvirus 1. Arch. Virol. 140, 1653–1662. doi: 10.1007/BF01322539 7487497

[B11] Ganeshpurkar A. Saluja A. K. (2017). The pharmacological potential of rutin. Saudi Pharm. J. 25, 149–164. doi: 10.1016/j.jsps.2016.04.025 28344465 PMC5355559

[B12] Garvey M. Suarez N. M. Kerr K. Hector R. Moloney-Quinn L. Arkins S.. (2018). Equid herpesvirus 8: Complete genome sequence and association with abortion in mares. PloS One 13, e0192301. doi: 10.1371/journal.pone.0192301 29414990 PMC5802896

[B13] Han Y. Ding Y. Xie D. Hu D. Li P. Li X.. (2015). Design, synthesis, and antiviral activity of novel rutin derivatives containing 1, 4-pentadien-3-one moiety. Eur. J. Medicinal Chem. 92, 732–737. doi: 10.1016/j.ejmech.2015.01.017 25618020

[B14] Han B. Li S. Lv Y. Yang D. Li J. Yang Q.. (2019). Dietary melatonin attenuates chromium-induced lung injury via activating the Sirt1/Pgc-1α/Nrf2 pathway. Food Funct. 10, 5555–5565. doi: 10.1039/C9FO01152H 31429458

[B15] Khan F. Pandey P. Upadhyay T. K. Jafri A. Jha N. K. Mishra R.. (2020). Anti-cancerous effect of rutin against HPV-C33A cervical cancer cells via G0/G1 cell cycle arrest and apoptotic induction. Endocr Metab. Immune Disord. Drug Targets 20, 409–418. doi: 10.2174/1871530319666190806122257 31385777

[B16] Kovacsics C. E. Gill A. J. Ambegaokar S. S. Gelman B. B. Kolson D. L. (2017). Degradation of heme oxygenase-1 by the immunoproteasome in astrocytes: A potential interferon-gamma-dependent mechanism contributing to HIV neuropathogenesis. Glia 65, 1264–1277. doi: 10.1002/glia.23160 28543773 PMC5739592

[B17] Lai X. Zhang Y. Wu J. Shen M. Yin S. Yan J. (2023). Rutin attenuates oxidative stress via PHB2-mediated mitophagy in MPP (+)-induced SH-SY5Y cells. Neurotox. Res. 41, 242–255. doi: 10.1007/s12640-023-00636-5 36738374

[B18] Lee Y. J. Choi J. H. Kang K. K. Sung S. E. Lee S. Sung M.. (2024). Antioxidant and antimelanogenic activities of lactobacillus kunkeei NCHBL-003 isolated from honeybees. Microorganisms 12, 188. doi: 10.3390/microorganisms12010188 38258014 PMC10818717

[B19] Li L. Hu X. Li S. Li Y. Zhao S. Shen F.. (2023). Cobalt protoporphyrin blocks EqHV-8 infection via IFN-alpha/beta production. Anim. (Basel) 13, 2690. doi: 10.3390/ani13172690 PMC10487175 37684954

[B20] Li L. Sun W. Hu Q. Wang T. Zhu G. Zhao Q.. (2022). Identification of MYH9 key domain involved in the entry of PRRSV into permissive cells. Front. Microbiol. 13, 865343. doi: 10.3389/fmicb.2022.865343 35694306 PMC9174932

[B21] Lin Y. J. Chang Y. C. Hsiao N. W. Hsieh J. L. Wang C. Y. Kung S. H.. (2012). Fisetin and Rutin as 3C protease inhibitors of enterovirus A71. J. Virol. Methods 182, 93–98. doi: 10.1016/j.jviromet.2012.03.020 22465253

[B22] Ling L. J. Lu Y. Zhang Y. Y. Zhu H. Y. Tu P. Li H.. (2020). Flavonoids from Houttuynia cordata attenuate H1N1-induced acute lung injury in mice via inhibition of influenza virus and Toll-like receptor signalling. Phytomedicine 67, 153150. doi: 10.1016/j.phymed.2019.153150 31958713

[B23] Liu C. Guo W. Lu G. Xiang W. Wang X. (2012). Complete genomic sequence of an equine herpesvirus type 8 Wh strain isolated from China. J. Virol. 86, 5407. doi: 10.1128/JVI.00445-12 22492929 PMC3347380

[B24] Loboda A. Damulewicz M. Pyza E. Jozkowicz A. Dulak J. (2016). Role of Nrf2/HO-1 system in development, oxidative stress response and diseases: an evolutionarily conserved mechanism. Cell. Mol. Life Sci. 73, 3221–3247. doi: 10.1007/s00018-016-2223-0 27100828 PMC4967105

[B25] Ma X. Ren X. Zhang X. Wang G. Liu H. Wang L. (2024). Rutin ameliorate PFOA induced renal damage by reducing oxidative stress and improving lipid metabolism. J. Nutr. Biochem. 123, 109501. doi: 10.1016/j.jnutbio.2023.109501 37890710

[B26] Mazik M. (2022). Promising therapeutic approach for SARS-CoV-2 infections by using a rutin-based combination therapy. ChemMedChem 17, e202200157. doi: 10.1002/cmdc.202200157 35489042 PMC9321678

[B27] Mihic D. Loinjak D. Maricic L. Smolic R. Sahinovic I. Steiner K.. (2022). The relationship between Nrf2 and HO-1 with the severity of COVID-19 disease. Medicina (Kaunas) 58, 1658. 36422196 10.3390/medicina58111658 PMC9693233

[B28] Miklasinska-Majdanik M. Kepa M. Wasik T. J. Zapletal-Pudelko K. Klim M. Wojtyczka R. D. (2023). The direction of the antibacterial effect of rutin hydrate and amikacin. Antibiotics (Basel) 12, 1469. doi: 10.3390/antibiotics12091469 37760765 PMC10525965

[B29] Mohamed E. K. Fathy M. M. Sadek N. A. Eldosoki D. E. (2024). The effects of rutin coat on the biodistribution and toxicities of iron oxide nanoparticles in rats. J. Nanoparticle Res. 26, 1–21. doi: 10.1007/s11051-024-05949-w

[B30] Negahdari R. Bohlouli S. Sharifi S. Maleki Dizaj S. Rahbar Saadat Y. Khezri K.. (2021). Therapeutic benefits of Rutin and its nanoformulations. Phytother Res. 35, 1719–1738. doi: 10.1002/ptr.6904 33058407

[B31] Ninfali P. Antonelli A. Magnani M. Scarpa E. S. (2020). Antiviral properties of flavonoids and delivery strategies. Nutrients 12, 2534. doi: 10.3390/nu12092534 32825564 PMC7551920

[B32] Perk A. A. Shatynska-Mytsyk I. Gercek Y. C. Boztas K. Yazgan M. Fayyaz S.. (2014). Rutin mediated targeting of signaling machinery in cancer cells. Cancer Cell Int. 14, 124. doi: 10.1186/s12935-014-0124-6 25493075 PMC4260193

[B33] Savov V. M. Galabov A. S. Tantcheva L. P. Mileva M. M. Pavlova E. L. Stoeva E. S.. (2006). Effects of Rutin and quercetin on monooxygenase activities in experimental influenza virus infection. Exp. Toxicol. Pathol. 58, 59–64. doi: 10.1016/j.etp.2006.05.002 16793246

[B34] Schvartz G. Edery N. Moss L. Hadad R. Steinman A. Karniely S. (2020). Equid herpesvirus 8 isolated from an adult donkey in Israel. J. Equine Vet. Sci. 94, 103247. doi: 10.1016/j.jevs.2020.103247 33077102

[B35] Sebastiano M. Chastel O. de Thoisy B. Eens M. Costantini D. (2016). Oxidative stress favours herpes virus infection in vertebrates: a meta-analysis. Curr. Zool. 62, 325–332. doi: 10.1093/cz/zow019 29491920 PMC5829443

[B36] Seminotti B. Grings M. Tucci P. Leipnitz G. Saso L. (2021). Nuclear factor erythroid-2-related factor 2 signaling in the neuropathophysiology of inherited metabolic disorders. Front. Cell. Neurosci. 15, 785057. doi: 10.3389/fncel.2021.785057 34955754 PMC8693715

[B37] Tonelli C. Chio I. I. C. Tuveson D. A. (2018). Transcriptional regulation by Nrf2. Antioxid. Redox Signal. 29, 1727–1745. doi: 10.1089/ars.2017.7342 28899199 PMC6208165

[B38] Wang T. Du Q. Niu Y. Zhang X. Wang Z. Wu X.. (2019). Cellular p32 is a critical regulator of porcine Circovirus type 2 nuclear egress. J. Virol. 93, 10–128. doi: 10.1128/JVI.00979-19 PMC6854514 31511386

[B39] Wang T. Hu L. Liu M. Wang T. Hu X. Li Y.. (2022a). The emergence of viral encephalitis in donkeys by Equid Herpesvirus 8 in China. Front. Microbiol. 13, 840754. doi: 10.3389/fmicb.2022.840754 35308333 PMC8930201

[B40] Wang T. Hu L. Wang Y. Liu W. Liu G. Zhu M.. (2022b). Identification of equine herpesvirus 8 in donkey abortion: a case report. Virol. J. 19, 10. doi: 10.1186/s12985-021-01738-2 34991640 PMC8734136

[B41] Wang T. Xi C. Yu Y. Liu W. Akhtar M. F. Li Y.. (2023). Characteristics and epidemiological investigation of equid herpesvirus 8 in donkeys in Shandong, China. Arch. Virol. 168, 99. doi: 10.1007/s00705-023-05704-x 36871102

[B42] Wu Y. Zhou S. Zhao A. Mi Y. Zhang C. (2023). Protective effect of Rutin on ferroptosis-induced oxidative stress in aging laying hens through Nrf2/HO-1 signaling. Cell Biol. Int. 47, 598–611. doi: 10.1002/cbin.11960 36378583

[B43] Yoon J. Park T. Kim A. Song H. Park B. J. Ahn H. S.. (2022). First report of equine parvovirus-hepatitis and equine hepacivirus coinfection in horses in Korea. Transbound Emerg. Dis. 69, 2735–2746. doi: 10.1111/tbed.14425 34919324

[B44] Zakaryan H. Arabyan E. Oo A. Zandi K. (2017). Flavonoids: promising natural compounds against viral infections. Arch. Virol. 162, 2539–2551. doi: 10.1007/s00705-017-3417-y 28547385 PMC7087220

